# Ileoileal intussusception induced by a gastrointestinal stromal tumor

**DOI:** 10.1186/1477-7819-6-133

**Published:** 2008-12-17

**Authors:** Kontantinos Vasiliadis, Evangelos Kogopoulos, Michael Katsamakas, Evangelos Karamitsos, Christos Tsalikidis, Byron Pringos, Andreas Tsalikidis

**Affiliations:** 1 Surgical Department, General Hospital of Kilkis, Nosokomiou 1, GR-61 100, Kilkis, Greece

## Abstract

**Background:**

Gastrointestinal stromal tumors are mesenchymal tumors of the gastrointestinal tract of varying malignant potential that are believed to originate from neoplastic transformation of the interstitial cells of Cajal. They may occur anywhere along the gastrointestinal tract, but most commonly arise in the stomach or small intestine. They usually grow exophytically invading adjacent organs or perforating into the peritoneal cavity. They may also cause bleeding or obstructive symptoms. Intussusception and obstruction is a very uncommon presentation of these lesions because of their tendency to grow in an exraluminal fashion.

**Case presentation:**

We present an unusual case of ileoileal intussusception in a 79-year-old female patient caused by a gastrointestinal stromal tumor located in the terminal ileum, and review the diagnostic and therapeutic approach highlighting the difficulty in diagnosing this entity preoperatively as a cause for intestinal obstruction.

**Conclusion:**

This case presents an unusual malignant cause of adult intussusception and highlights the importance of computed tomography scanning in the accurate diagnosis of this rare entity.

## Background

Gastrointestinal stromal tumors (GISTs) are the least common of small intestinal malignant neoplasms, with an annual incidence of 1.2 cases per million population [1-3]. Their distribution in the small intestine indicates that 17.7% are in the duodenum, 47.6% in the jejunum, and 34.7% in the ileum [1,2]. They typically present with an abdominal mass, pain, or surgical emergencies such as bleeding and obstructive symptoms [3] Intussusception and obstruction is a very uncommon presentation of these lesions because of their tendency to grow in an extraluminal fashion. Besides, adult intussusception represents only about 1% to 5% of all cases of intestinal obstructions and is commonly caused by a lesion acting as the apex of intussusception [4].

We present an unusual case of ileoileal intussusception in a 79-year-old female patient caused by a GIST located in the terminal ileum, acting as the apex of intussusception, and review the diagnostic and therapeutic approach highlighting the difficulty in diagnosing this entity preoperatively as a cause for intestinal obstruction.

## Case presentation

A 79-year-old woman presented with a 5-day history of colicky pain in the right lower abdominal quadrant, variable in severity, aggravated by food ingestion, and associated with nausea and abdominal distension. She also acknowledged new onset of constipation and vomiting. Past medical history included total gastrectomy, seven years before this admission, for a stage IA gastric adenocarcinoma. Ever since she had been followed-up annually and she remained free of disease in good health, except of cobalamin deficiency anemia for which she was taking high-dose oral mecobalamin supplementation. She also had an 8-year history of type II diabetes mellitus and a 10-year history of arterial hypertension.

On physical examination she was pale, in no acute distress, with mild tachycardia and normal blood pressure. The abdomen was moderately distended, with tenderness on deep palpation, in the right lower quadrant. All hernial orifices were normal and there was no evidence of incisional hernia at the gastrectomy scar. No significant weight loss or palpable mass was identified. Auscultation revealed hypoactive bowel sounds and digital rectal examination showed an empty rectal vault. Gynecological examination was unremarkable.

**Figure 1:**
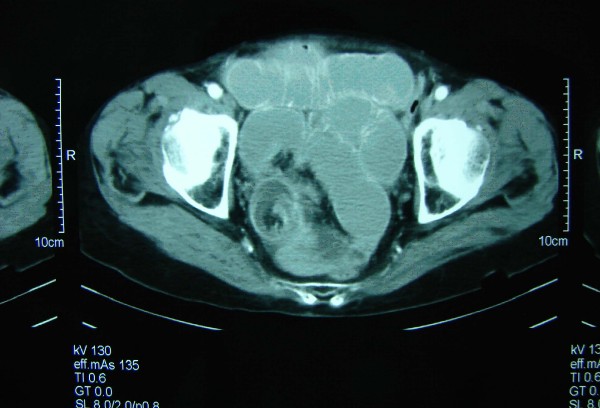
Abdominal computed tomography scan showing the characteristic "target sign", establishing the diagnosis of intussusception.

**Figure 2:**
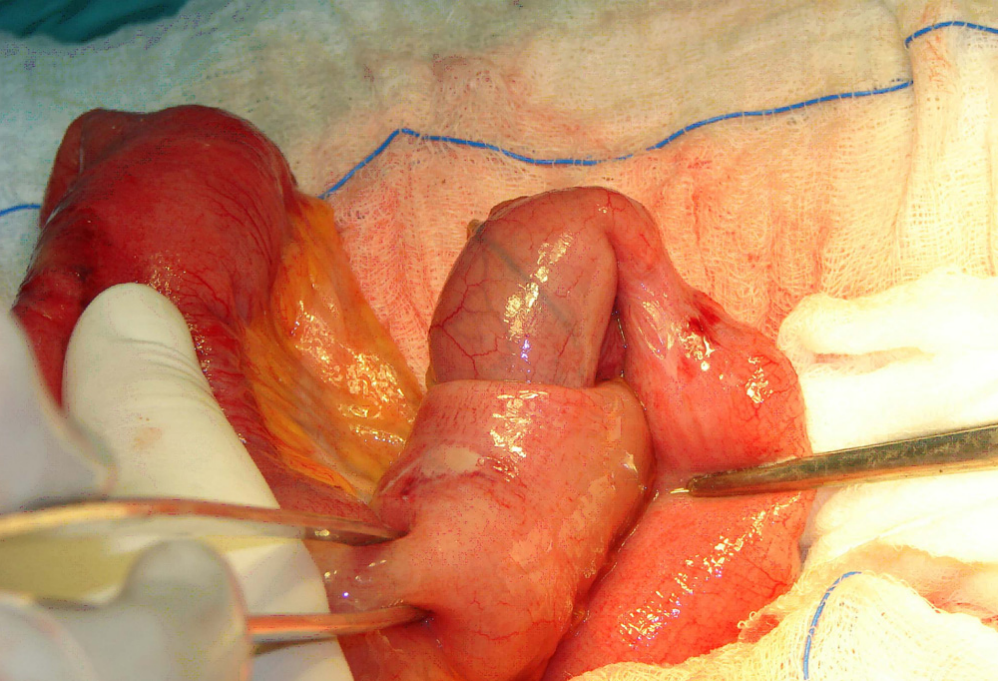
Intraoperative photograph. Gentle reduction of the ileoileal intussusception

**Figure 3:**
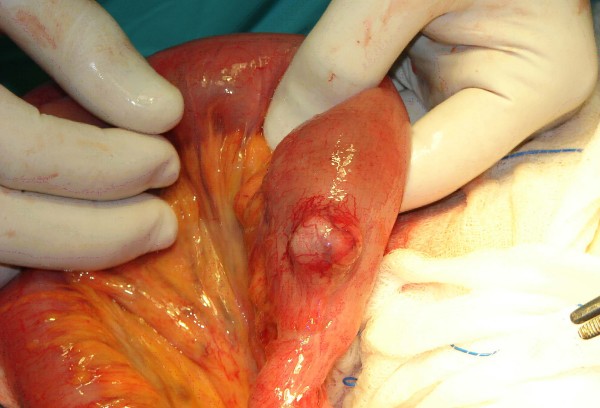
Intraoperative photograph. The small bowel gastrointestinal stromal tumor which acted as the apex of intussusception, exposed after gentle reduction of the intussusceptum.

Laboratory analysis showed leukocytosis (13.4 × 10^9^L^-1^) and anemia (hemoglobin 9.2 mg/dl). Hepatic and renal function values in addition to urinary tests were within normal limits. A plain abdominal film showed multiple air fluid levels. Computed tomographic (CT) scan of the abdomen with oral contrast showed the "target" sign of intussusception in the right lower quadrant of the abdomen (Figure 1), following the CT scan, the patient's pain and abdominal distension deteriorated and led to an emergent exploratory laparotomy. This revealed an ileoileal intussusception (Figure 2) secondary to a 2.2 × 1.8 × 2 cm intramural mass in the terminal ileum, located 20 cm proximal to the ileocecal valve (Figure 3). The intussuscepted intestinal segments were obstructing the lumen, causing dilatation in the intestine before the intussusception. Further intraoperative exploration did not reveal any other pathological findings. An end-to-end ileoileal anstomosis was fashioned after gentle reduction and resection of the neoplastic segment; wide mesenteric lymphadenectomy was also performed. The patient made a very satisfactory recovery and was discharged after 7 days. Eleven months after surgery she is doing well.

The pathology report confirmed that the neoplasm was a small bowel GIST. The margins of surgical resection and all identified mesenteric lymph nodes were negative for malignancy. The tumor cells were pleomorphic with 7–8 mitosis in 50 high power fields. Immunohistochemical studies showed positive stains for protein S100, vimentin and c-kit and negative for desmin, actin, and CD 34. The tumor was estimated to have an intermediate malignant potential and the patient is under surveillance according to the guidelines of the European Society of Medical Oncology [5].

## Discussion

The small intestine accounts for little more than 1% of all gastrointestinal malignancies being remarkably resistant to the development of both benign and malignant tumors [6,7]. Malignant neoplasms of small bowel include adenocarcinoma, carcinoid, lymphoma, endocrine tumors, metastases and GISTs [3]. The latter represent the most common mesenchymal tumor of the gastrointestinal tract, accounting for approximately 13% of all small intestinal malignancies [7]. Notwithstanding this, GISTs are the least common of small intestinal malignant neoplasms and because of their insidious presentation, they are often not suspected prior to surgery. Consequently, their diagnosis is often delayed or even overlooked and usually is made after laparotomy and formal pathologic examination [3].

Small bowel GISTs are usually asymptomatic, especially in their early stages and they often go unrecognized until severe symptoms ensue, which can create surgical emergencies [3,7]. Although slow-growing, GISTs can grow very large before producing signs and symptoms, as they tend to displace adjacent structures without invasion [8]. In addition, they can spread to the liver, lungs, and bones via the bloodstream, bypassing the local lymph nodes [9]. They are often detected incidentally on physical examination, radiologic imaging, endoscopy, or laparotomy, but eventually the majority of patients develop symptoms because of disease progression [10].

Symptomatic GISTs often present with non-specific and vague abdominal symptoms and signs [11] The most common clinical findings include an abdominal mass, pain, bleeding, weight loss, nausea, vomiting and obstructive ileus [3,8]. These symptoms mainly depend on the size and the location of the tumor, with lesions distal to the ligament of Treitz having a tendency to present with either obstruction or bleeding [12]. GISTs tend to grow in an extraluminal fashion; however, they can also erode into the lumen of the gastrointestinal tract inducing significant hemorrhage or anemia from occult bleeding [4]. They can also rupture into the peritoneal cavity causing significant hemorrhage [13]. In addition to symptoms from mass effect or bleeding, GISTs can cause intussusception or rarely intestinal obstruction [14]. In this case the patient was asymptomatic until intestinal obstruction developed. Of note is that a very rare and relatively small in diameter tumor acted as a lead point for the ileoileal intussusception, which is also a very uncommon condition.

Intussusception accounts for only 1% to 5% of all cases of intestinal obstruction in adults and is rarely diagnosed preoperatively [15,16]. This is mainly related to the paucity of patients and the non-specific complaints and physical findings of intussusception that can be confused with other causes of intestinal obstruction. Common physical findings include abdominal distension and tenderness, an abdominal mass, colicky pain, nausea, vomiting, change in bowel habits, constipation, hypoactive to absent bowel sounds, and bleeding [17]. The classic triad of abdominal mass, tenderness, and haemoglobin-positive stools is rarely found and was not present in this case [18]. Furthermore, in 70% to 90% of adult cases, the intussusception has an identifiable lead lesion, and is more likely to occur in the small intestine. In this case the tumor was located extraluminally at the terminal ileum acting as the apex of intussusception, while the intussuscepted intestinal segments completely obstructed the small bowel lumen. These facts lead to the hypothesis that the presence of a submucosal lesion such as a GIST altered normal bowel peristalsis and acted as the leading point in the intussusceptum. The subsequent peristaltic activity of the bowel produced an area of constriction above the stimulus and relaxation below, thus invaginating the leading point (intussusceptum) through the distal part of the terminal ileum lumen (intussuscipiens). The patient had also a low haemoglubin value. Intussusception may cause gastrointestinal bleeding because of ischemia and necrosis of the tumor; however, in this case the anemia had macrocytic indices and was due to cobalamin deficiency, secondary to the previous gastrectomy.

Because of the non-diagnostic physical findings of intussusception, most patients undergo further investigation with various imaging modalities. An ideal diagnostic algorithm has to be defined; however, CT scanning has been reported to be the most useful tool for the diagnosis of intestinal intussusception, and it appears to be superior to other contrast studies, ultrasonography, or endoscopy [19,20]. Furthermore, as the majority of adult intussusception is caused by an underling neoplastic lesion, abdominal CT should probably be the first imaging investigation upon suspicion of intussusceptions, and can provide additional staging information. The density of the intussusceptum within the lumen of the intussuscipien gives the characteristic "target sign" or "sausage shaped appearance", [18], which was present in this case.

Surgical resection is recommended in nearly all cases of adult intussusception, because of the high prevalence of structural anomalies and the relatively high risk of underlying malignancy. However, the issue of reduction versus mandatory primary resection remains a topic of some controversy. Weilbacher and associates [21] established the principle of mandatory primary resection without reduction, because of the high incidence of underlying malignancy. They also claimed that reduction includes the theoretical risk of intraluminal seeding or venous embolization in regions of ulcerated mucosa [21]. On the other hand, mandatory resection necessitates the excision of a long segment of small bowel, which may compromise the mesenteric vessels. Therefore it has been proposed that gentle operative reduction, when feasible, can be attempted safely before resection, to avoid the unnecessary excision of a healthy bowel [22]. In this case a gentle reduction was attempted successfully, resulting in the preservation of small bowel length without compromising the oncological extent of the resection.

## Conclusion

This case presents an unusual malignant cause of adult intussusception and highlights the importance of computed tomography scanning in the accurate diagnosis of this rare entity.

## Consent

Written informed consent was obtained from the patient for publication of this case report and accompanying images. A copy of the written consent is available for review by the Editor-in-Chief of this journal. Additionally, the Scientific Council of General Regional Hospital of Kilkis gave its assent for the publication of data in medical Journal.

## Competing interests

The authors declare that they have no competing interests.

## Authors' contributions

KV conceived the study, performed the surgical management, study design, and prepared the final version of the manuscript. EK participated in literature search, study design and in the surgical management. MK participated in study design, literature search, and preparation of the manuscript. CT participated in the revision of the study and preparation of its final version. EK revised the manuscript for scientific content. BP participated in literature search, and in the surgical management. AT has given the approval for submitting the final version of the manuscript.
